# Long-term efficacy and safety of guselkumab in Chinese patients with moderate-to-severe plaque psoriasis

**DOI:** 10.3389/fmed.2023.1285972

**Published:** 2023-12-14

**Authors:** Jianfeng Zheng, Wenjuan Chen, Xuemei Yi, Ning Yu, Yangfeng Ding, Yunlu Gao

**Affiliations:** ^1^Department of Dermatology, Shanghai Skin Disease Hospital, Tongji University School of Medicine, Shanghai, China; ^2^Institute of Psoriasis, Tongji University School of Medicine, Shanghai, China

**Keywords:** Chinese patients, efficacy, guselkumab, plaque psoriasis, real-life, safety

## Abstract

**Background:**

Randomized controlled trials indicated guselkumab, the first anti-interleukin-23 monoclonal antibody, is efficacious in plaque psoriasis. However, guselkumab's performance in real life is scarcely examined, especially in China.

**Objectives:**

This work aimed to assess the long-term effectiveness of guselkumab in actual clinical practice in China.

**Methods:**

A retrospective study was performed for plaque psoriasis cases administered guselkumab in Shanghai Skin Disease Hospital between January 2020 and September 2022.

**Results:**

A total of 37 patients were included (29 men, 78.4%), with a mean follow-up period of 72.3 ± 26.7 weeks (range of 12–108 weeks). At baseline, clinical examination revealed a mean PASI of 12.3 ± 7.1, a mean BSA of 17.1 ± 18.1, and a mean DLQI of 7.7 ± 4.3. Twenty-two (62.9%) and 17 (48.6%) cases achieved PASI 90 and PASI 100 responses at week 28. From weeks 60 to 92, >80% of cases achieved PASI 90 and PASI 100 responses. Regarding safety, no cases of serious AEs were recorded. A total of nine cases (24.3%) had different abnormal results in *HBV markers*, and two were T-SPOT positive. There was no hepatitis B virus or tuberculosis outbreak in these patients.

**Conclusion:**

This real-life study confirmed the long-term efficacy and safety of guselkumab in daily clinical practice.

## 1 Introduction

Guselkumab represents an anti-interleukin (IL)-23 monoclonal antibody that reduces inflammation in psoriatic lesions by directly inhibiting IL-23/Th17 signaling (1). It is approved for the treatment of moderate-to-severe plaque psoriasis in cases eligible for systemic therapy or phototherapy (2). In a 48-week phase III, randomized, double-blind, placebo-controlled, active-comparator trial, the safety and efficacy of guselkumab combined with adalimumab or placebo were assessed in adults with moderate-to-severe plaque psoriasis. At week 16, 85.1% of cases administered guselkumab achieved an Investigator Global Assessment (IGA) of 0/1, and 65.9 and 6.9% in the adalimumab and placebo groups, respectively (*P* < 0.001). Psoriasis Activity Severity Index 90 (PASI 90) was achieved at week 16 in 73.3% of cases administered guselkumab, and 49.7 and 2.9% in the adalimumab and placebo groups, respectively (*P* < 0.001) (3). In real life, a retrospective study assessed patients with moderate-to-severe psoriasis administered guselkumab in the Psoriasis Care Center of Dermatology at the University Federico II of Naples between June 2019 and December 2021 (4). A total of 44 patients were included (28 men, 63.6%; mean age of 59.0 ± 10.2 years). Significantly improved PASI and body surface area (BSA) were detected at each follow-up (PASI decreased from 13.9 ± 8.1 to 0.9 ± 0.7 at week 52, and BSA decreased from 24.3 ± 19.6 to 1.3 ± 1.4, both *p* < 0.001). Only three (6.8%) cases discontinued guselkumab treatment for secondary inefficacy. No cases of serious adverse events were recorded. Real-life studies seem to confirm guselkumab's efficacy and safety in a real-world setting, while cases in China have been rarely assessed (5).

Thus, we carried out a single-center, retrospective trial to assess the long-term efficacy and safety of guselkumab in Chinese patients, and some of whom were previously administered one or more biologic agents in a real-life setting.

## 2 Materials and methods

A retrospective study was carried out to assess patients with moderate-to-severe psoriasis administered guselkumab in the Psoriasis Care Center of Dermatology at the Shanghai Skin Diseases Hospital between January 2020 and July 2022. Inclusion criteria were (a) diagnosis of moderate-to-severe plaque psoriasis by a dermatologist for at least 6 months and (b) guselkumab administration for at least 12 weeks.

For each case, the following data were collected at baseline: age, sex, psoriasis duration, BSA, PASI, Dermatology Life Quality Index (DLQI), body mass index (BMI), comorbidities, family history of psoriasis, and past and current psoriasis therapies.

At each follow-up visit (weeks 4, 12, 28, 44, 60, 76, and 92) psoriasis severity indexes (PASI and BSA) were assessed as well as adverse events (AEs). At baseline and follow-up, routine blood tests [blood count with formula, transaminases, creatinine, azotemia, glycaemia, erythrocyte sedimentation rate, C reactive protein, total cholesterol and triglycerides levels, protein electrophoresis, Hepatitis B Virus (HBV) markers, anti-Hepatitis C Virus (HCV) markers, and mycobacterium tuberculosis T cell enzyme-linked immunospot tuberculous test (T-SPOT)] were collected. This study followed the Declaration of Helsinki, and all patients provided signed informed consent.

## 3 Statistical analysis

Statistical analysis was carried out to examine the statistical significance of clinical improvement. Clinicodemographic data were analyzed using descriptive statistics. Continuous data are mean ± standard deviation, and categorical variables are number and proportion. The Mann-Whitney *U*-test and the *t*-test were performed to compare values obtained at different times during treatment for categorical and continuous variables, respectively. *P* < 0.05 was considered statistically significant. GraphPad Prism 8.0 was used for data analysis.

## 4 Results

A total of 40 patients administered guselkumab in our department were enrolled. A final number of 37 (92.5%) cases (29 men, 78.4%) were included in the study (Table 1). Mean patient age was 44.1 ± 10.6 years (range of 24–65 years), and the mean disease duration was 16.8 ± 10.5 years. A family history of psoriasis was found in 8.1% of cases (3/37). A total two patients (5.4%) also had psoriatic arthritis. Regarding comorbidities, the most common were *abnormal HBV markers* (9, 24.3%), followed by obesity (7, 18.9%), T-SPOT positive (2, 5.4%), dyslipidemia (2, 5.4%), hypertension (1, 2.7%), diabetes (1, 2.7%), hyperuricemia (1, 2.7%), and abnormal kidney function (1, 2.7%) (Table 1). A total of 23 cases (62.2%) had previous treatment with at least one conventional systemic treatment, including methotrexate, cyclosporine, and narrow band (Nb)-ultraviolet B (UVB) phototherapy, while 12 (32.4%) had previous exposure to one or more biologic agents. A total of nine (24.3%) patients had failed anti-tumor necrosis factor-α (anti-TNFα), four (10.3%) had failed secukinumab, and two (5.4%) had failed ustekinumab (Table 1).

**Table 1:** Baseline patient data.

	**History of biological treatments**		
	**Total**	**Biological treatments**	**No biological treatments**
Total number of patients (F/M)	37 (8/29)	12 (2/10)	25 (6/19)
Age (Mean SD, years)	44.1 ± 10.6	45.2 ± 8.5	43.6 ± 11.6
Disease course (Mean SD, years)	16.8 ± 10.5	18 ± 10.9	16.7 ± 10.6
Involvement of the scalp, *n* (%)	21 (56.8%)	8 (66.7%)	13 (52%)
Involvement of nails, *n* (%)	20 (54.1%)	8 (66.7%)	12 (48%)
History of PsA, *n* (%)	2 (5.4%)	1 (8.3%)	1 (4%)
Family history of psoriasis, *n* (%)	3 (8.1%)	2 (16.7%)	1 (4%)
PASI score (Mean SD)	12.3 ± 10.6	13.4 ± 8.2	11.8 ± 6.6
BSA score (Mean SD)	17.1 ± 18.1	21.1 ± 20.5	15.3 ± 16.9
DLQI score (Mean SD)	7.7 ± 7.3	8.3 ± 4.7	7.4 ± 4.2
**BMI**, ***n*** **(%)**			
Underweight	1 (2.7%)	1 (8.3%)	0 (0%)
Normal weight	20 (54.1%)	6 (50%)	14 (56%)
Overweight	14 (37.8%)	5 (41.7%)	9 (36%)
Obese	2 (5.4%)	0 (0%)	2 (8%)
**Comorbidities**, ***n*** **(%)**			
*Positive HBV markers*	9 (24.3%)	4 (33.3%)	5 (20%)
Positive T-SPOT	2 (5.4%)	1 (8.3%)	1 (4%)
Dyslipidemia	2 (5.4%)	2 (16.7%)	0 (0%)
Hypertension	1 (2.7%)	0 (0%)	1 (4%)
Diabetes	1 (2.7%)	0 (0%)	1 (4%)
Hyperuricemia	1 (2.7%)	1 (8.3%)	0 (0%)
Abnormal kidney function	1 (2.7%)	1 (8.3%)	0 (0%)
**Previous treatments**, ***n*** **(%)**			
Acitretin	11 (29.7%)	4 (33.3%)	7 (28%)
Methotrexate	10 (27%)	4 (33.3%)	6 (24%)
UB-UVB	11 (29.7%)	3 (25%)	8 (32%)
Etanercept	2 (5.4%)	2 (16.7%)	
Adalimumab	4 (10.8%)	4 (33.3%)	
Infliximab	6 (16.2%)	6 (50%)	
Secukinumab	4 (10.8%)	4 (33.3%)	
Ustekunumab	2 (5.4%)	2 (16.7%)	
Ixekizumab	1 (2.7%)	1 (8.3%)	

BMI, body mass index; PASI, Psoriasis Area and Severity Index; BSA, body surface area; DLQI, dermatology life quality index; HBV, hepatitis B virus; SD, standard deviation; PsA, psoriatic arthritis; UB-UVB, Narrow Band-UVB; T-SPOT, mycobacterium tuberculosis T cell enzyme-linked immunospot tuberculous test.

**Figure 1:**
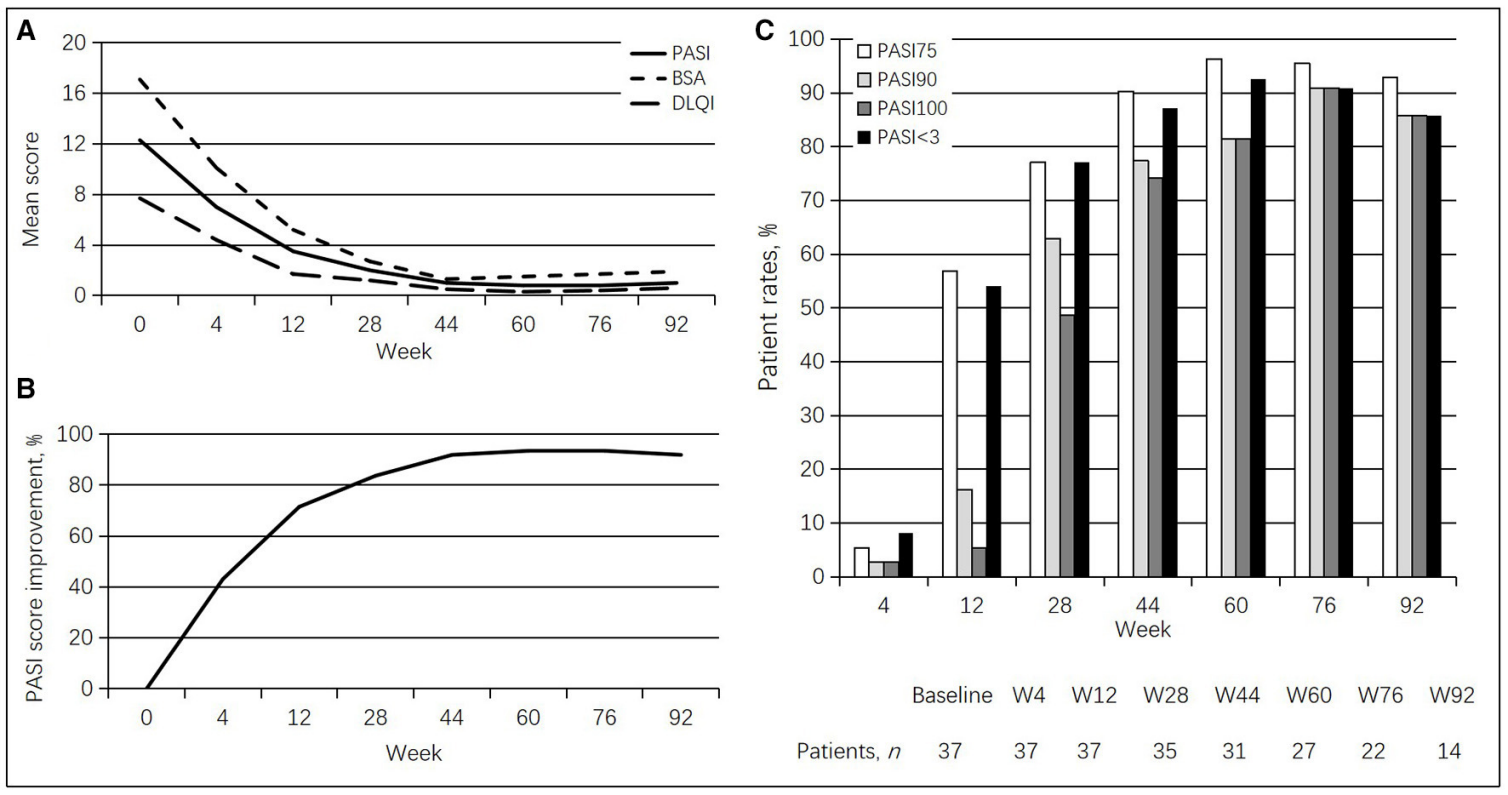
Guselkumab's efficacy in psoriasis patients. **(A)** Changes in mean PASI, BSA, and DLQI scores. **(B)** Percentages of mean PASI score improvement. **(C)** Rates of patients achieving PASI 75, PASI 90, and PASI 100 responses at different times.

At baseline, clinical examination revealed a mean PASI of 12.3 ± 7.1, a mean BSA of 17.1 ± 18.1, and a mean DLQI of 7.7 ± 4.3. Regarding PASI, a statistically significant improvement in PASI value was detected at week 4 (7.0 ± 4.9, *p* < 0.001), with a PASI score improvement of 43.1%. At week 12, a PASI 75 response was achieved by 21 (56.8%) patients, with PASI < 3.0 in 20 (54.1%) subjects. From weeks 44 to 92, >90% of patients had achieved a PASI 75 response. Especially, at weeks 60 and 76, a PASI 75 response was found in 96.3 and 95.5% of cases, respectively. At week 28, a PASI < 3.0 response was achieved by 27 (77.1%) patients. From weeks 44 to 92, >85% of patients had achieved a PASI < 3.0 response. Similarly, at weeks 60 and 76, a PASI < 3.0 response was found in 92.6 and 90.9% of cases, respectively. A total of 22 (62.9%) and 17 (48.6%) patients reached PASI 90 and PASI 100 responses at week 28. PASI 90 and PASI 100 responses were found in >80% of cases between weeks 60 and 92. At week 76, a total of 20 (90.9%) patients had achieved PASI 90 and PASI 100 responses. A statistically significant improvement was also found for BSA at each follow-up (10.1 ± 11.0 at week 4, *p* < 0.001; 2.7 ± 5.6 at week 28, *p* < 0.0001; 1.5 ± 6.9 at week 60, *p* < 0.0001; up to 1.9 ± 6.4 at week 92, *p* < 0.0001) and DLQI at each follow-up (4.4 ± 3.3 at week 4, *p* < 0.001; 1.2 ± 2.6 at week 28, *p* < 0.0001; 0.3 ± 1.2 at week 60, *p* < 0.0001; 0.6 ± 1.7 at week 92, *p* < 0.0001). All psoriasis index results are detailed in Table 2 and Figure 1.

**Table 2:** Psoriasis assessment at baseline and weeks 4, 12, 28, 44, 60, 76, and 92.

**Week**	**0**	**4**	**12**	**28**	**44**	**60**	**76**	**92**
PASI	12.3 ± 7.1	7.0 ± 5.0	3.5 ± 3.8	2.0 ± 3.7	1.0 ± 2.4	0.8 ± 2.8	0.8 ± 3.0	1 ± 3.0
		*p* < 0.05	*p* < 0.001	*p* < 0.0001	*p* < 0.0001	*p* < 0.0001	*p* < 0.0001	*p* < 0.0001
BSA	17.1 ± 18.1	5.2 ± 7.2	5.2 ± 7.2	2.7 ± 5.7	1.4 ± 6.0	1.52 ± 7.2	1.4 ± 5.6	1.4 ± 5.6
		*p* < 0.05	*p* < 0.05	*p* < 0.0001	*p* < 0.0001	*p* < 0.0001	*p* < 0.0001	*p* < 0.0001
DLQI	7.7 ± 7.3	1.7 ± 2.0	1.7 ± 2.0	1.2 ± 2.6	0.3 ± 1.2	0.3 ± 1.4	0.4 ± 1.4	0.4 ± 1.4
		*p* < 0.001	*p* < 0.001	*p* < 0.0001	*p* < 0.0001	*p* < 0.0001	*p* < 0.0001	*p* < 0.0001

PASI, Psoriasis Area and Severity Index; BSA, body surface area; DLQI, dermatology life quality index.

Till now, 11 patients (29.7%) have interrupted the treatment. One case was lost to follow-up at week 20, one needed chemotherapy and interrupted the treatment at week 36, two voluntarily withdrew from the treatment at week 44, one was operated on and had to end the treatment at week 68, and one left to study abroad and stopped the treatment at week 68. A PASI 100 response was found in 83.3% of cases between weeks 20 and 36. However, the patient who lost follow-up had achieved a PASI 90 response at week 12. Another five patients interrupted the treatment between weeks 28 and 52 due to inefficacy. One patient with previously failed treatment with TNF inhibitors and ixekizumab discontinued the treatment at week 28, and three cases previously administered TNF inhibitors discontinued the treatment between weeks 44 and 52 (Table 3). A PASI 75 response was achieved by two cases at week 20, indicating an ineffective rate of 13.5% in this study.

**Table 3:** Detailed information from some ineffective patients.

**No**.	**Patient 1**	**Patient 2**	**Patient 3**	**Patient 4**	**Patient 5**	**Patient 6**	**Patient 7**
Sex	Male	Male	Female	Female	Male	Female	Male
Age (years)	48	40	45	36	35	40	35
Disease course (years)	22	29	27	26	21	14	12
Involvement of the scalp	Y	Y	N	N	N	Y	N
Involvement of nails	Y	Y	N	N	N	Y	N
Family history of psoriasis	N	N	Y	N	N	N	N
BMI	29.4	22.9	20.8	19.8	16.3	32.7	25.2
*HBV markers*	Anti-HBe(+) Anti-HBc(+)	N	N	N	N	N	N
Positive T-SPOT	N	N	N	N	N	N	N
Previous systemic	ACI/MTX/	ACI/MTX/	INX	N	INX	N	N
Treatments	ADA/INX	ADA/SEC/IXE					
BSA score (Week 0)	50%	14%	6%	7%	6%	70%	10%
DLQI score (Week 0)	14	5	10	6	6	16	7
PASI score Week 0	31.4	6.8	11	6.8	7.2	35	12
Week 12	18	2.4	10	3.4	3	16	7
Week 20	15	12	11	3.4	1.5	13	5
Week 28	16	UST	11	IXE	1.5	10.7	5
Week 44	Interrupt		SEC		5.5	12	3
Week 52					UST	13.6	3
Week 84						11	3

ACI, acitretin; ADA, adalimumab; BMI, body mass index; BSA, body surface area; DLQI, dermatology life quality index; HBV, hepatitis B virus; INX, infliximab; IXE, ixekizumab; MTX, methotrexate; PASI, Psoriasis Area and Severity Index; T-SPOT, mycobacterium tuberculosis T cell enzyme-linked immunospot tuberculous test; SEC, secukinumab; UST, ustekinumab.

**Figure 2:**
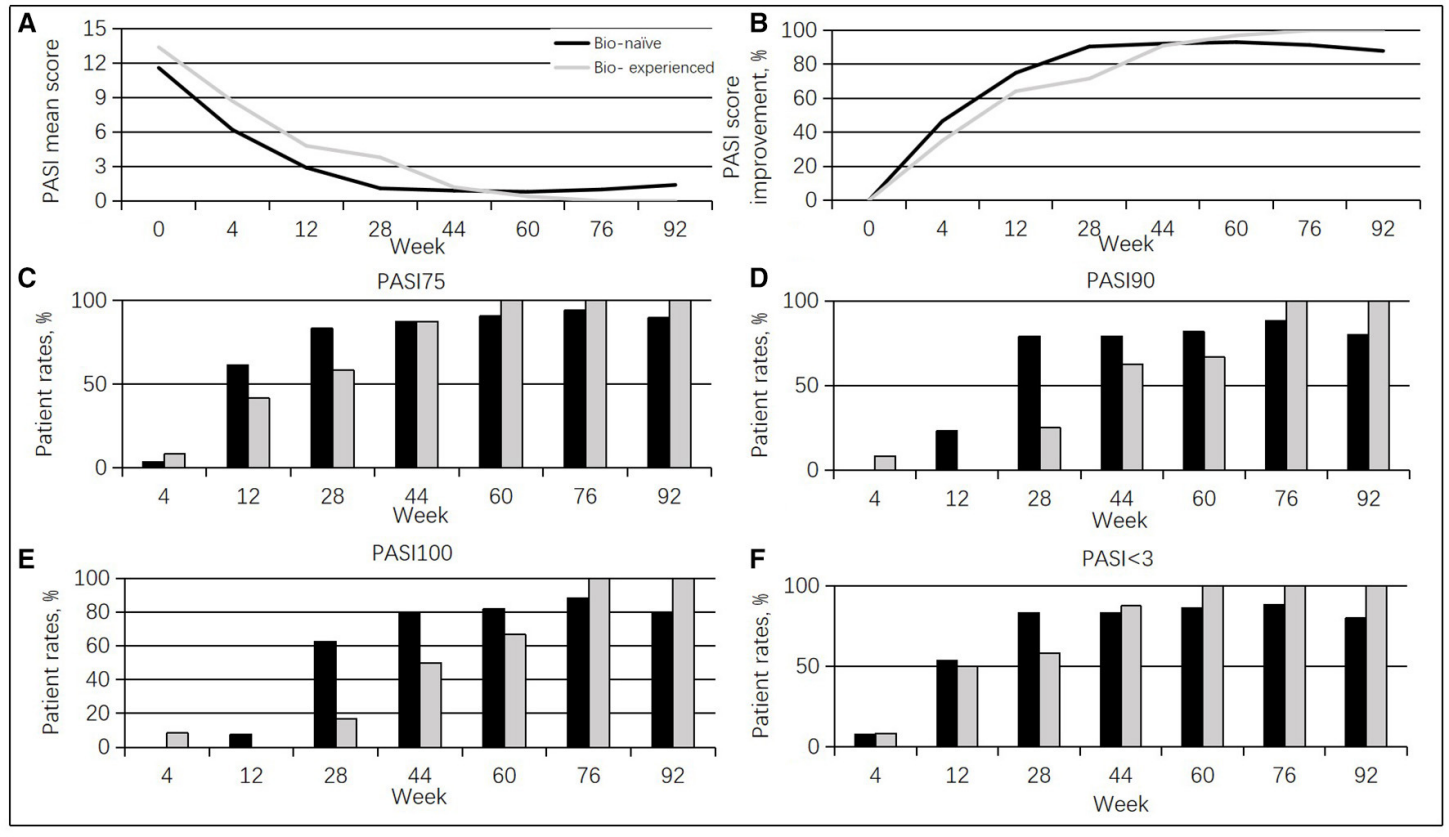
Guselkumab's efficacy between the bio-naïve and bio-experienced cases. **(A)** Changes in mean PASI scores. **(B)** Percentages of mean PASI score improvement. **(C)** Rates of cases achieving a PASI 75 response at different times. **(D)** Rates of cases achieving a PASI 90 response at diverse times. **(E)** Rates of cases achieving a PASI 100 response at diverse times. **(F)** Rates of cases achieving a PASI < 3.0 response at diverse times.

Moreover, bio-experienced cases (patients who have had any biologics input) (10 men, 83.3%; mean age of 45.2 ± 8.5 years, range of 35–65 years) showed a PASI superior to the bio-naïve cases (patients who have not had any biologics input) (19 men, 76%; mean age of 43.6 ±11.6 years, range of 24–65 years) at baseline (13.4 vs. 11.6, *p* = 0.485). From weeks 4 to 28, bio-experienced cases had less pronounced PASI score improvement than the bio-naïve group (35.1 vs. 46.6%, at week 4; 64.2 vs. 75%, at week 12; 71.6 vs. 90.5%, at week 28). However, no differences were detected between the two groups after week 28. Additionally, less bio-experienced patients achieved PASI 75 and PASI < 3.0 than the bio-naïve cases at weeks 12 and 28, and differences in PASI 90 and PASI 100 were found at weeks 12, 28, 44, and 60 (Figure 2). In this study, the ineffective rate was higher in the bio-experienced patients (33.3%) compared with bio-naïve patients (4%).

**Figure 3:**
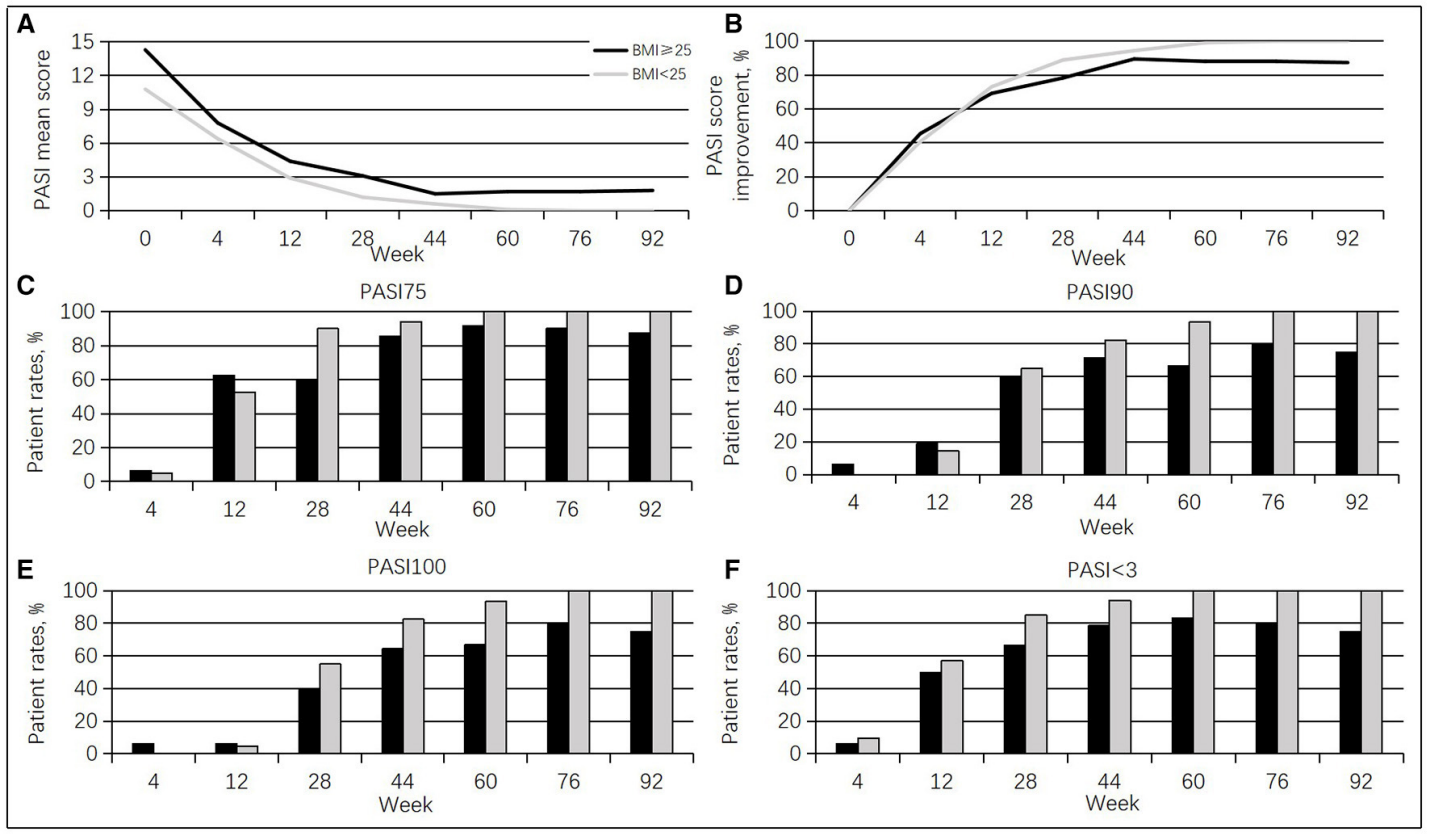
Guselkumab's efficacy between the BMI ≥ 25 group and the BMI < 25 groups. **(A)** Changes in mean PASI scores. **(B)** Percentages of mean PASI score improvement. **(C)** Rates of cases achieving a PASI 75 response at different times. **(D)** Rates of cases achieving a PASI 90 response at diverse times. **(E)** Rates of cases achieving a PASI 100 response at diverse times. **(F)** Rates of cases achieving a PASI < 3.0 response at diverse times.

Furthermore, this study population had high body mass index values (overweight + obese > 43.2%). Cases with BMI ≥ 25 group (15 men, 93.8%; mean age of 42.3 ± 10.4 years, range of 24–61 years) showed higher PASI scores than those with BMI < 25 group (14 men, 66.7%; mean age of 45.5 ± 10.8 years, range of 33–61 years) at baseline (14.3 vs. 10.8, *p* = 0.167). From weeks 12 to 92, cases with a BMI < 25 had more pronounced PASI score improvement compared with the BMI ≥ 25 group. The BMI < 25 group achieved PASI 75, PASI 90, PASI 100, and PASI < 3.0 responses to a greater extent than the BMI ≥ 25 group from weeks 28 to 92 (Figure 3).

Regarding safety, nine patients (24.3%) had different abnormal results in *HBV markers at baseline*. A total of five cases were anti-HBc positive; two were anti-HBe and anti-HBc positive; one was HBsAg and anti-HBc positive; and one was HBsAg, anti-HBe, and anti-HBc positive. HBV-DNA was normal at baseline. At present, except for one patient who discontinued the treatment for inefficacy, the remaining patients have completed the follow-up. A total of two cases were T-SPOT positive. However, they had no history of tuberculosis or abnormal manifestations of chest computed tomography. There was no hepatitis B or tuberculosis outbreak during follow-up. No cases of serious AEs, injection site reactions, candida infection, malignancy, or cardiovascular events were reported.

## 5 Discussion

Biologic application in psoriasis has improved beyond expectations in the therapeutic response in cases with moderate-to-severe psoriasis, with excellent safety features (6). PASI 90 and PASI 100 responses are considered the treatment goals in moderate-to-severe psoriasis, suggesting complete or nearly complete clearance, generally related to significant improvement in health-related quality of life. As a human monoclonal antibody targeting the p19 subunit of IL-23, guselkumab was the first antibody of its class to be approved for treating moderate-to-severe plaque psoriasis in adults (7–10). In this study, guselkumab was administered as a 100-mg subcutaneous injection at weeks 0 and 4, and then at 8-week intervals. The current patients had a mean PASI of 12.3 ± 7.1 and a mean disease duration of 16.8 ± 10.5 years at baseline, which corroborated the mean baseline PASI of 13.7 with an average evolution time for their psoriasis of 20 years reported by Rodriguez Fernandez-Freire L et al. in Spain (11). This study achieved a PASI 75 response below that reported in a previous study (12 weeks); differences were also detected in PASI 90 and PASI 100 responses at week 12 between the two populations. However, there were no differences in PASI 75, PASI 90, or PASI 100 responses following 36 weeks of treatment. Differences in guselkumab's efficacy at the early stage might depend on regions, but more data are required for confirmation. Moreover, a previous study population had a high BMI (overweight + obese > 89.1%) (11). In the present study, the ratio was 43.2%. However, no relationship between treatment effectiveness and weight was found. In this work, patients had a mean follow-up period of 72.3 ± 26.7 weeks (range of 12–108 weeks). The results suggested that guselkumab has excellent long-term effectiveness.

Furthermore, there were 12 cases with previously failed treatment with other biologic agents in this study; we found that less bio-experienced cases achieved a PASI 75 response than bio-naïve cases at 12 and 28 weeks. A total of five patients interrupted the treatment for inefficacy, including four who had previous treatment with other biologic agents. One case with previously failed treatment with both TNF inhibitors and ixekizumab discontinued the treatment at week 28; meanwhile, three cases previously administered TNF inhibitors discontinued the treatment at weeks 44–52. Similarly, Hung et al. (12) reported that previously administered anti-IL-17 cases had less substantial PASI improvement than biologic-naïve cases at the early stage, suggesting that biologic treatment may reduce guselkumab's effectiveness. However, Bonifati et al. (13) examined 9 patients who switched to guselkumab upon anti-IL-17 failure, demonstrating a significant PASI improvement after 3 months of treatment. In a short-term trial of 55 patients (11), 27 cases previously administered anti-IL-17 still had confirmed guselkumab's effectiveness. In addition, guselkumab discontinuation for inefficacy was found in only one patient. Finally, the effectiveness and safety of guselkumab were examined in 44 patients with previously failed anti-IL-17 treatment in a real-life setting up to 52 weeks (4). Significantly improved PASI [PASI decreased from 13.9 ± 8.1 to 0.9 ± 0.7 (*p* < 0.001) at week 52] and BSA [BSA from 24.3 ± 19.6 to 1.3 ± 1.4 (*p* < 0.001)] were detected, and only three cases (6.8%) discontinued the treatment for secondary inefficacy. These data indicated biologic exposure could not influence guselkumab's efficacy.

The safety of guselkumab was confirmed since no cases of serious AEs were reported. In this study, nine cases had different abnormal results in the *HBV markers*, and two cases were T-SPOT positive. However, there was no hepatitis B or tuberculosis outbreak in the examined patients. In agreement, randomized controlled trials and real-life studies have reported that guselkumab has an excellent safety profile (4, 14, 15). The most common adverse event was infection, mostly respiratory tract infections. No patients discontinued the treatment for AE in this study.

The specific pathogenesis of psoriasis remains undefined, although the IL-23/IL-17 axis is considered to play an important role (1). Particularly, IL-23 is a dimer comprising a specific subunit, p19, and a p40 subunit, which is also found in IL-12. These cytokines may activate two T-cell types, including helper T-cell types 1 and 17, which release psoriatic cytokines such as IL-17, interferon-γ, TNF-α, and IL-22 (16, 17). This may explain why some patients previously administered anti-IL-17 still showed an excellent response to guselkumab. Certainly, further investigation is required to help the clinician select the best treatment option based on the patient's particularities, ensuring the highest odds of achieving and maintaining expected clinical outcomes.

## Data availability statement

The original contributions presented in the study are included in the article/supplementary material, further inquiries can be directed to the corresponding authors.

## Ethics statement

The studies involving humans were approved by the Institutional Review Board of Shanghai Skin Disease Hospital. The studies were conducted in accordance with the local legislation and institutional requirements. The participants provided their written informed consent to participate in this study.

## Author contributions

JZ: Conceptualization, Data curation, Formal analysis, Investigation, Writing—original draft, Writing—review & editing. WC: Data curation, Formal analysis, Writing—review & editing. XY: Resources, Writing—review & editing. NY: Resources, Writing—review & editing. YD: Funding acquisition, Resources, Writing—review & editing. YG: Project administration, Resources, Supervision, Validation, Writing—review & editing.
